# CT and MR Unilateral Brain Features Secondary to Nonketotic Hyperglycemia Presenting as Hemichorea-Hemiballism

**DOI:** 10.1155/2016/5727138

**Published:** 2016-05-09

**Authors:** Víctor Manuel Suárez-Vega, Carlos Sánchez Almaraz, Ana Isabel Bernardo, Ricardo Rodríguez-Díaz, Ana Díez Barrio, Leticia Martín Gil

**Affiliations:** ^1^Radiology Department, Hospital Infanta Elena, Valdemoro, 28342 Madrid, Spain; ^2^Neurology Department, Hospital Infanta Elena, Valdemoro, 28342 Madrid, Spain

## Abstract

Hemichorea-hemiballism is an unusual hyperkinetic movement disorder characterized by continuous involuntary movements of an entire limb or both limbs on one side of the body. The acute onset of this disorder occurs with an insult in contralateral basal ganglia. Ischemic events represent the most common cause. Nonketotic hyperglycemia comes in second place. Nonketotic hyperglycemic hemichorea-hemiballism (NHH) is a rare cause of unilateral brain abnormalities on imaging studies confined to basal ganglia (mainly putaminal region as well as caudate nucleus). Subtle hyperdensity in striatal region can be found on CT studies whereas brain MR imaging typically shows T1 hyperintensity and T2 hypointensity in the basal ganglia contralateral to the movements. Diagnosis is based on both glucose levels and neuroimaging findings. Elevated blood glucose and hemoglobin A1c levels occur with poorly controlled diabetes. In this case report, our aim is to present neuroimaging CT and MR unilateral findings in an elderly woman secondary to nonketotic hyperglycemia presenting as hemichorea-hemiballism.

## 1. Introduction

Hemichorea-hemiballism is an unusual hyperkinetic movement disorder characterized by continuous involuntary movements of an entire limb or both limbs on one side of the body. These movements are mostly irregular, variable in amplitude, and with no recognizable pattern [1].

Bedwell first described hemichorea-hemiballism associated with hyperglycemia in 1960. He reported the case of a 57-year-old woman with hemiballism that resolved as the blood glucose levels were normalized [2].

It was Yahikozawa et al. who first reported 3 diabetic patients with the unique combination of hemiballism and striatal hyperintensity on T1-weighted MRI as a new syndrome in 1994 [3].

NHH is most frequently reported in elderly patients, typically Asian, who have type II diabetes. The majority of cases published in the literature involve female patients [4]. Although this syndrome usually occurs in patients with previously known diabetes, it could also be an initial manifestation of diabetes [5].

## 2. Case Presentation

A 57-year-old female patient from East Europe was admitted in the emergency room of our institution with chest pain. Her serum glucose and hemoglobin A1c levels were 800 mg/dL and 15.9%, respectively. There were no ketones in the serum. Serum glucose was treated by insulin continuous perfusion and the patient was discharged.

A few hours later, she was readmitted presenting involuntary and violent movements on the left side of the body.

**Figure 1:**
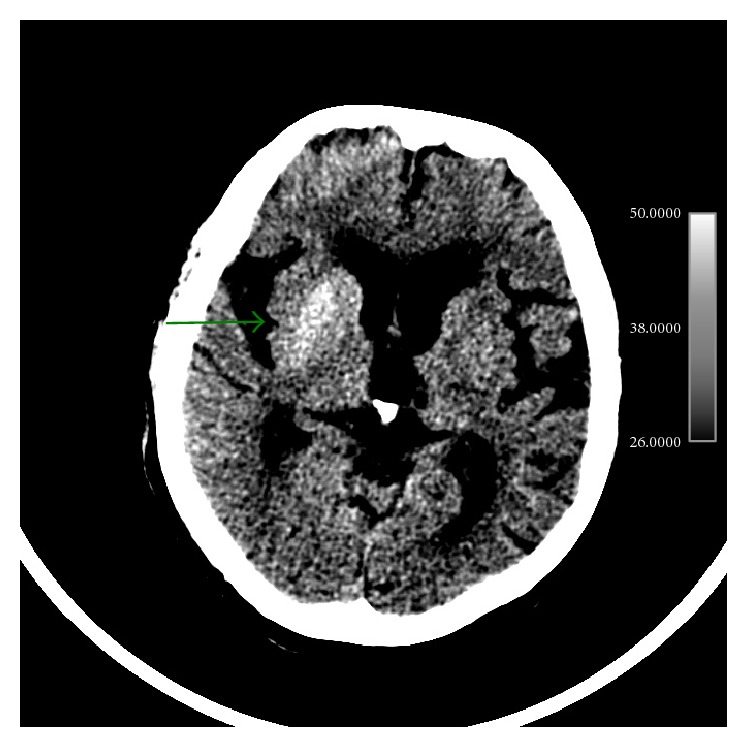
Findings: unenhanced axial brain CT depicts hyperdense striatum (caudate nucleus and putamen). This finding is better seen using narrow window width and higher center settings.

An unenhanced cranial CT was performed to rule out an ischemic event. Findings were initially misdiagnosed, reporting hypoattenuation of left basal ganglia instead of considering an abnormal increased attenuation on right basal ganglia. Using narrow window width and slightly higher center settings better depicted this finding (Figure 1).

As correction of hyperglycemia did not lead to improvement of the involuntary movements, the patient was admitted in the Intensive Care Unit and an aggressive intravenous therapy was performed. Initially, risperidone up to 2 mg/dL and a continuous perfusion of clonazepam was administrated, with no response. Further treatment with tetrabenazine 25 mg twice a day was necessary for improvement of the movements in the next few days.

**Figure 2:**
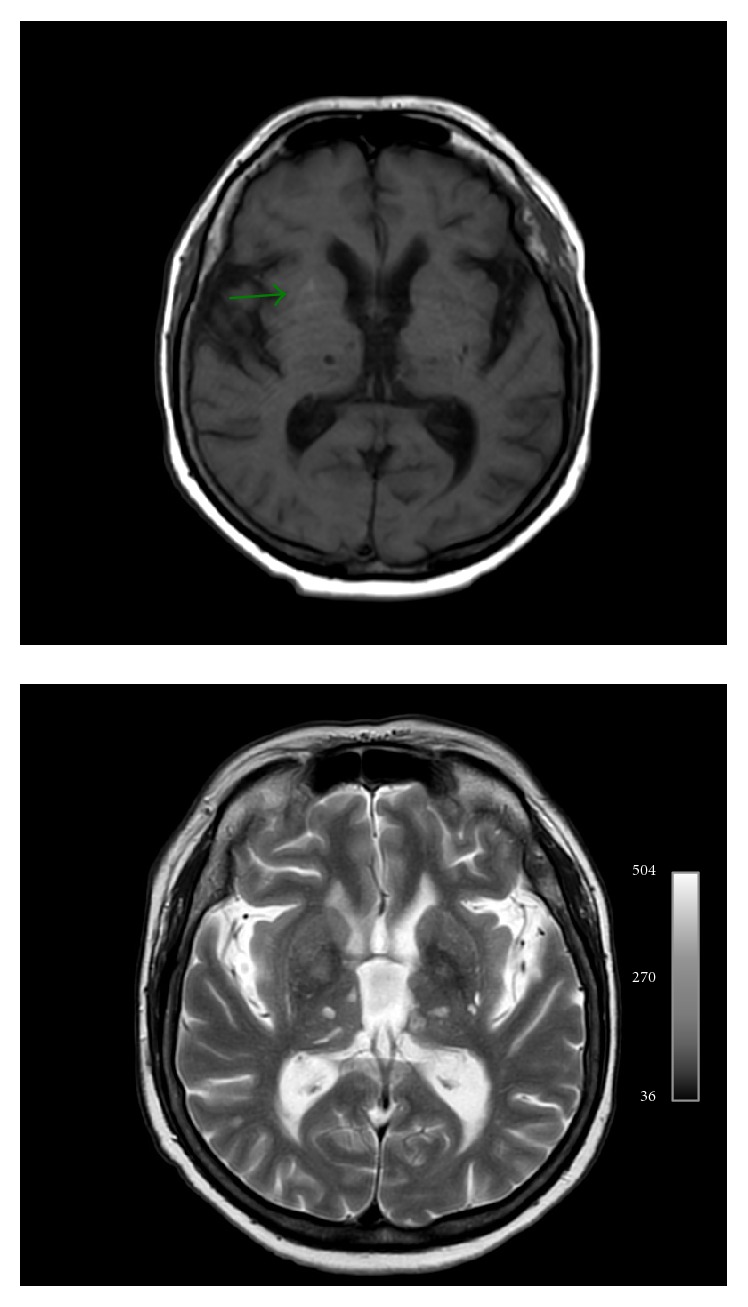
Axial T1-weighted sequence depicts right basal ganglia hyperintensity with unremarkable signal abnormality on axial T2-weighted.

**Figure 3:**
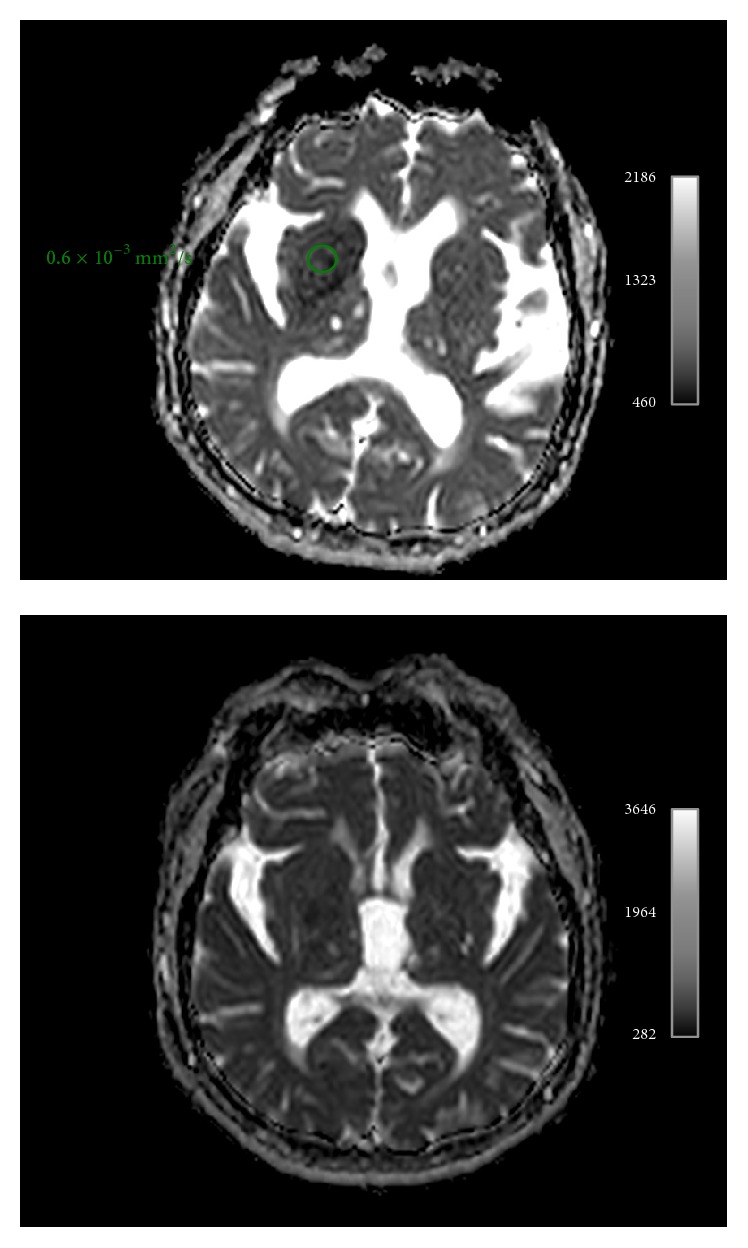
ADC map shows slight decreased apparent diffusion coefficient in right basal ganglia. Symmetrical normal ADC values one month later.

MR imaging of the brain was performed 2 days after the second admission showing right basal ganglia hyperintensity on T1-weighted sequences and fairly normal signal intensity on T2-weighted and FLAIR sequences (Figure 2). The apparent diffusion coefficients (ADCs) were slightly lower in right basal ganglia compared to those on the left side. ADC values were symmetrical on both basal ganglia (Figure 3).

**Figure 4:**
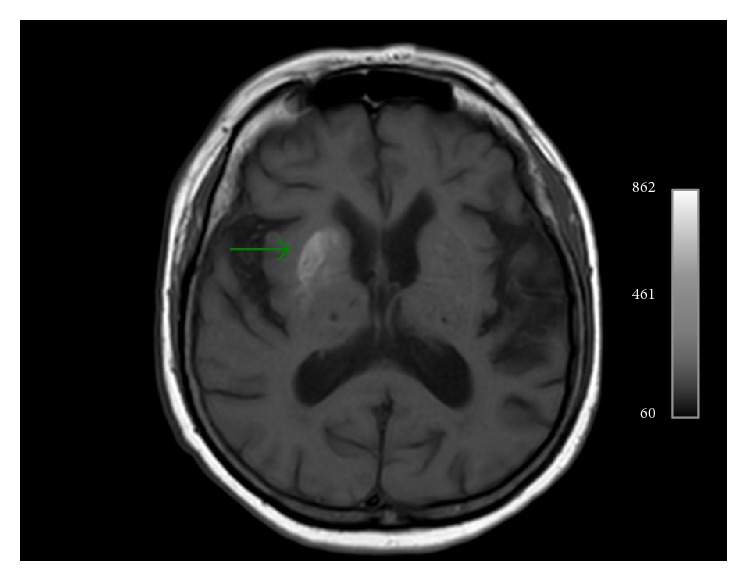
Striking increase of T1-weighted signal intensity within right striatum one month after the onset of symptoms. Patient was asymptomatic when this MR study was performed.

One month later—movement disorders completely recovered—the patient was reimagined, depicting a striking increase on T1-weighted signal intensity, whereas the signal intensity of the rest of the sequences was within normal limits (Figure 4).

**Figure 5:**
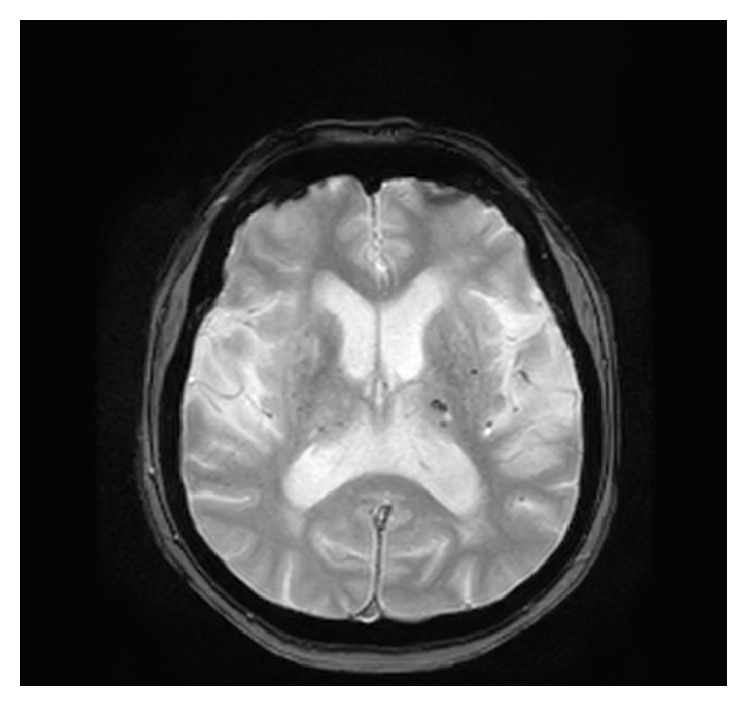
Axial T2 gradient echo sequence shows no signal abnormality within right striatum but some scattered blooming artifacts within left thalami consistent with petechial hemorrhages.

**Figure 6:**
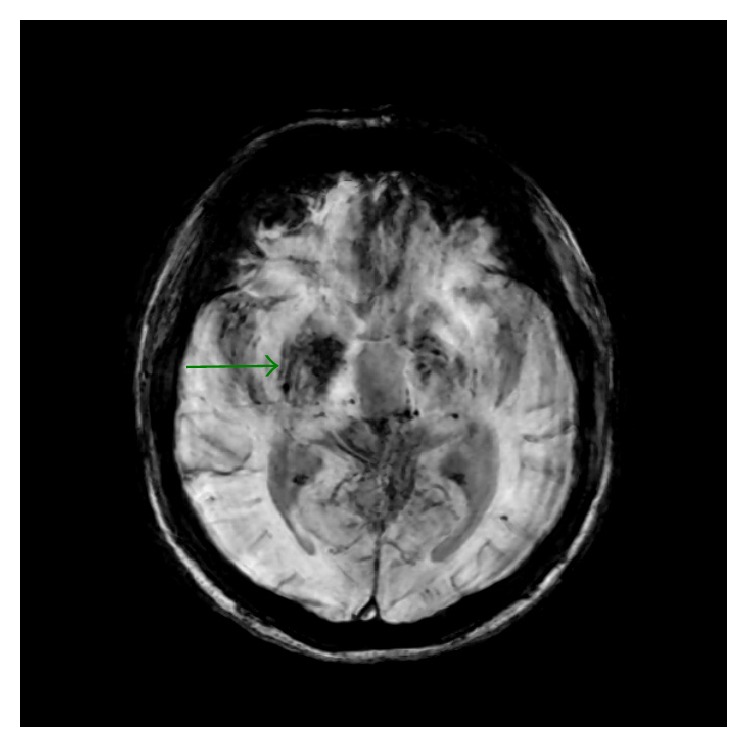
Axial minimum intensity projection of susceptibility weighted imaging at 10-month follow-up depicts slightly hypointense signal within right striatum.

Both MR studies performed with one month difference did not show any abnormality within right striatum on T2 gradient echo sequences (Figure 5). However, a 10-month follow-up MR scan showed hypointensity within right striatum on susceptibility weighted images (SWI) (Figure 6).

**Figure 7:**
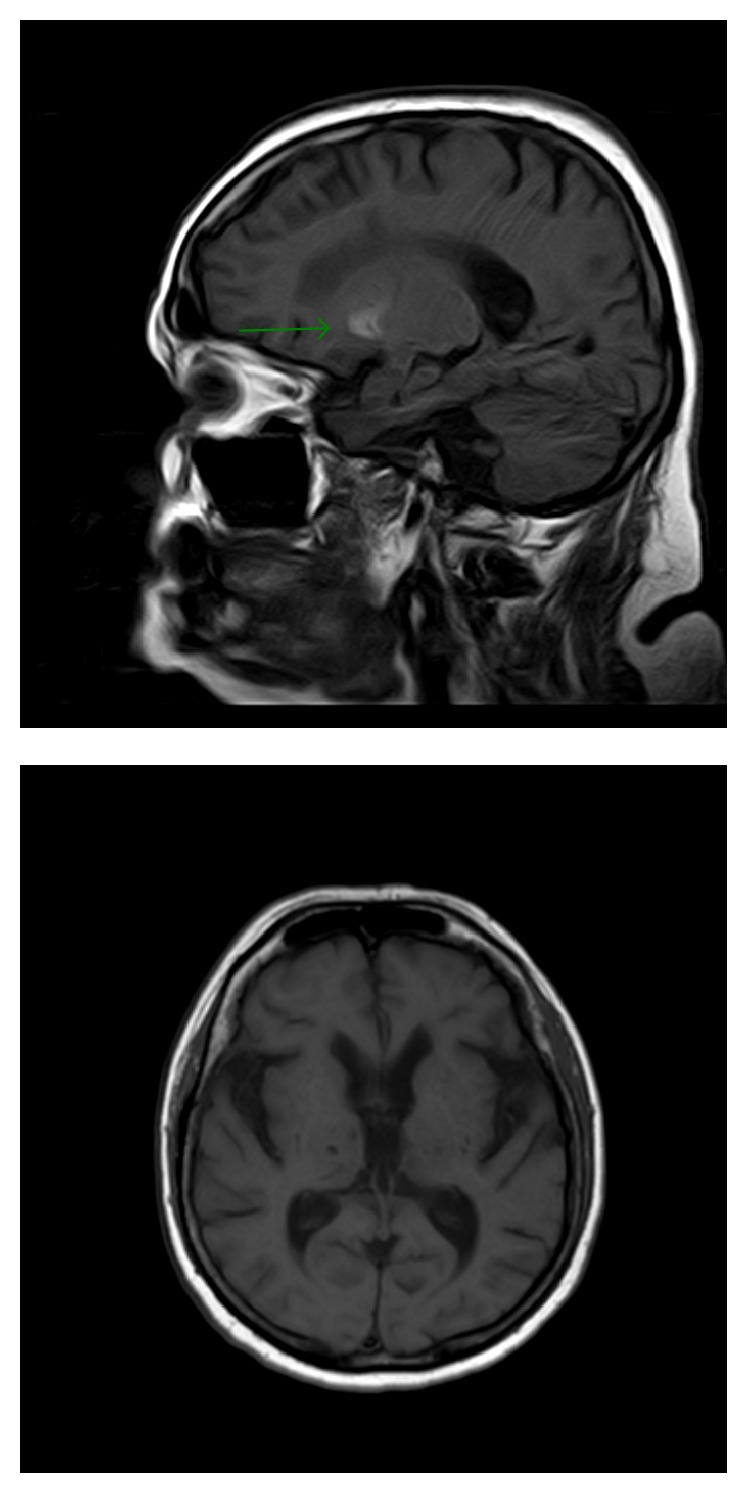
4-month and 10-month follow-up MR studies. Sagittal T1 depicts persistent hyperintensity within right striatum (green arrow), whereas a 10-month follow-up MR shows complete regression of signal abnormalities.

Several follow-up MR studies have been performed with an elapsed time of 4 months and 10 months. Whereas the 4-month follow-up study still depicted right basal ganglia hyperintensity, the 10-month MR study finally confirmed a total regression of signal abnormalities, showing a normal appearance right striatum. No signs of striatum atrophy were shown (Figure 7).

## 3. Discussion

The underlying pathophysiology of imaging changes described in NHH is poorly understood.

Some mechanisms include blood hyperviscosity caused by hyperglycemia, leading to blood brain barrier disruption; increased sensitivity of dopaminergic receptors in postmenopausal period, remember female predominance; decreased gamma-aminobutyric acid (GABA) in striatum secondary to a nonketotic state.

In addition, acute infarct, petechial hemorrhage, myelinolysis, and calcium deposition have also been postulated [6, 7]. MR findings suggest that petechial hemorrhage is less plausible since if the high signal on T1 corresponds with methemoglobin it should also be high signal on T2. This is not reported in our case or in previous cases [7]. We could not find any alterations on T2 gradient echo images involving right striatum. However, the 10-month follow-up scan showed slightly hypointense signal within right striatum on SW images. To our knowledge, there is only one series of three cases describing SWI findings in NHH syndrome [8]. These authors explain T1 hyperintensity from the protein hydration layer inside the cytoplasm of swollen gemistocytes appearing after an acute cerebral injury. These astrocytes also express metallothionein with zinc, which is thought to be the cause of asymmetric hypointensity of the posterior putamen on SWI.

Other authors suggest that high T1 signal could be related to manganese accumulation on gemistocytes [9].

The differential diagnosis of conditions presenting with hemichorea includes ischemic or hemorrhagic stroke, vasculitis, central nervous system lupus, mass lesions, multiple sclerosis, thyrotoxicosis, and drugs (such as neuroleptics or levodopa) [10].

However, clinical history and laboratory tests with striking increase of glucose levels make all these diagnoses unlikely.

Other causes to increased T1 signal in the basal ganglia are hypertensive hemorrhages, basal ganglia calcifications, Tay-Sachs disease, and tuberous sclerosis. A lenticulostriate isolated ischemic stroke can lead to a mismatch effect of false high signal on putaminal region.

Other metabolic or toxic disorders showing T1 signal increase include chronic hepatic encephalopathy, the most frequent, manganese toxicity during long-term parenteral nutrition, postcardiac arrest encephalopathy, hypoglycemic coma, hypothyroidism, neurofibromatosis, Fahr disease, Wilson disease, and carbon monoxide poisoning. In all these conditions a bilateral and symmetrical involvement is usually present [11].

Clinical course in patients with NHH is usually favorable and symptoms tend to resolve spontaneously within normalization of glucose levels. In addition, striatal high signal intensity may show resolution within a variable period of 2–11 months. In our case, T1 findings showed not only persistency but also increase of the signal whereas the other sequences partially resolved. We may emphasize that a period of 1 month separated both studies. Further follow-up studies confirmed resolution of findings in a period of 10 months, as reported previously in the literature.

One possible explanation could be acute putaminal dysfunction, secondary to hyperglycemic or hyperosmolar insult. This functional alteration could be associated with some degree of Wallerian degeneration of the internal white matter of the putamen. Protein desiccation occurring in the course of Wallerian degeneration could explain the CT hyperattenuation and the MR and DW imaging patterns in the early phase, as well as the variable evolution of imaging features with time [12]. In our patient, no signs of atrophy affecting right striatum were found in follow-up studies after the disappearance of high intensity signals on T1 sequences. This might be explained by the early improvement in hemichorea-hemiballism, as suggested by the meta-analysis of Oh et al. [4].

In addition, we were not able to find an explanation for the increased signal on T1 sequences after one month onset. Some studies with larger number of cases report an increased T1 signal on follow-up MR scans compared to baseline CT images, with a posterior resolution of MR abnormalities [11].

## 4. Conclusion 

NHH is a unique clinical-radiological entity with peculiar CT and MRI findings.

An early recognition is mandatory in order to establish the correct therapy (normalization of glucose levels) and avoid unnecessary treatments.
